# Hepatocyte nuclear factor 4α regulates megalin expression in proximal tubular cells

**DOI:** 10.1016/j.bbrep.2018.11.010

**Published:** 2018-12-12

**Authors:** Shota Sasaki, Ayami Hara, Masakiyo Sakaguchi, Masaomi Nangaku, Yusuke Inoue

**Affiliations:** a Division of Molecular Science, Graduate School of Science and Technology, Gunma University, Kiryu, Gunma 376-8515, Japan; b Department of Cell Biology, Okayama University Graduate School of Medicine, Dentistry and Pharmaceutical Sciences, Okayama 700-8558, Japan; c Division of Nephrology and Endocrinology, the University of Tokyo Hospital, Tokyo 113-8655, Japan; d Gunma University Center for Food Science and Wellness, Maebashi, Gunma 371-8510, Japan

**Keywords:** HNF4α, hepatocyte nuclear factor 4α, CKD, chronic kidney disease, PTECs, proximal tubular epithelial cells, Hepatocyte nuclear factor 4α, Proximal tubule, Kidney, Megalin

## Abstract

Hepatocyte nuclear factor 4α (HNF4α) is a member of the nuclear receptor superfamily and upregulates expression of many genes in the liver, pancreas, small intestine, and colon. HNF4α is also highly expressed in proximal tubular epithelial cells (PTECs) in kidney. PTECs reabsorb various substances through transporters, ion channels, and receptors, but the target genes for HNF4α in PTECs have not been investigated in detail. In the present study, we aimed to identify novel HNF4α target genes that are highly expressed in PTECs. Expression of many solute carrier transporter genes was upregulated by HNF4α in human PTEC-derived HK-2 cells. Notably, expression of megalin (*LRP2*), an endocytic receptor of various molecules involved in development and progression of chronic kidney disease (CKD), was strongly induced by HNF4α, and the transactivation potential of the megalin promoter was dependent on HNF4α expression. Moreover, HNF4α was found to directly bind to an HNF4α binding site near the transcription start site in the megalin gene. These results indicate that HNF4α plays an important role in maintaining reabsorption and metabolism in PTECs by positive regulation of several solute carrier transporter and megalin genes at the transcriptional level.

## Highlights

• HNF4α upregulates expression of several SLC transporters that are highly expressed in proximal tubular epithelial cells.
• HNF4α upregulates expression of magalin, multifunctional endocytic receptor in human proximal tubular epithelial cells.
• Transactivation of megalin gene is dependent on HNF4α expression and an HNF4α binding site in the megalin promoter.

## 1 Introduction

Hepatocyte nuclear factor 4α (HNF4α), an orphan member of nuclear receptor superfamily, is highly expressed in epithelial cells in liver, pancreas, small intestine, colon, and kidney, and HNF4α is essential for maintenance of the homeostasis in these tissues [1], [2]. HNF4α binds to approximately 40% of the promoter region of the genes expressed in human hepatocytes and pancreatic islets [3], indicating that HNF4α is a strong regulator in the liver and pancreatic. Since *Hnf4a* null-mice results in embryonic lethality [4], tissue-specific *Hnf4a*-null mice were generated in hepatocytes, pancreatic β cells, and intestinal epithelial cells. Based on many studies using liver-specific *Hnf4a*-null mice, hepatic HNF4α was found to be a central regulator for hepatocyte differentiation and function though direct regulation of many liver-specific genes [5], [6], [7], [8], [9]. In addition, pancreatic β cell-specific *Hnf4a*-null mice impaired glucose-stimulated insulin secretion [10]. Mutations in HNF4α gene related to maturity-onset diabetes of the young 1 (MODY1) [11], suggesting that pancreatic β cell-specific *Hnf4a*-null mice exhibited the similar phenotype as MODY1. Also, intestinal epithelial cell-specific *Hnf4a*-null mice were liable to get inflammatory bowel disease (IBD) [12]. Thus, HNF4α plays an essential role in maintenance of specific function in these tissues, but kidney-specific *Hnf4a*-null mice have not generated yet.

In kidney, HNF4α is highly expressed in proximal tubular epithelial cells (PTECs) [13], [14]. The proximal tubule is a part of the nephron of the kidney, and the main function of the proximal tubule is reabsorption of many substances such as water, glucose, potassium and sodium ions, phosphate, amino acids, and proteins filtrated in glomerulus. PTECs play an important role in renal injure and repair in acute kidney injury (AKI) and chronic kidney disease (CKD) progression [15], [16], [17]. Many transporters such as solute carrier (SLC) transporters and ATP-binding cassette transporters are expressed in the proximal tubule to reabsorb and excrete biomolecules. For example, sodium glucose cotransporter 2 (SGLT2), as known SLC5A2, is expressed in the proximal tubule, and *Sglt2*-deficient mice exhibit glycosuria and decreased glucose reabsorption [18]. Thus, many SGLT2 inhibitors are on the market for type 2 diabetic medications. Moreover, several studies were reported that HNF4α regulates expression of the transporters. Expression of *Slc6a19* gene, the neutral amino acid transporter in enterocytes, is transactivated by HNF4α and HNF1α [19]. HNF4α was also shown to bind to the promoter regions of *Slc22a1*, *Slc22a6*, and *Slc22a8* genes in rat kidney [20], indicating that HNF4α could play a central regulator of the transporters in the proximal tubule. However, the detailed regulation of the proximal tubule-enriched genes including the transporters by HNF4α remains poorly unexplained.

In the present study, we investigated to identify novel HNF4α target genes that are highly expressed in PTECs by overexpression of HNF4α in human HK-2 cells that express many characteristics of PTECs [21]. We found that expression of many transporters was induced by HNF4α, but expression of megalin (known as *LRP2*), a receptor for a large number of ligands, was strongly induced by HNF4α. Promoter activity of megalin gene was dependent on an HNF4α binding site and HNF4α expression, and HNF4α directly bound to the HNF4α binding site. Because megalin is involved in CKD development and progression, these finding may contribute to a detailed understanding of CKD development and progression through direct regulation of megalin expression by HNF4α.

## 2 Experimental procedures

### 2.1 Cell culture

HK-2 and HEK293T cells were cultured at 37 °C in Dulbecco's modified Eagle's medium (Wako) containing 10% fetal bovine serum (HyClone) and 100 units/ml penicillin/streptomycin (Thermo Fisher Scientific).

### 2.2 Construction of HNF4α expression plasmid and transient transfection

Full-length of human HNF4α cDNA was cloned into EcoRI and BamHI sites of pCMViR [22] and pEBMulti-Hygro vectors (Wako). pCMViR/HNF4α or empty plasmids were transfected into HK-2 cells cells using Fugene HD (Roche) and HEK293T cells using polyethyleneimine Max (Polyscience) as transfection reagents. After 48 h, the cells were harvested with Isogen II (Wako).

### 2.3 RNA extraction, reverse-transcription, and real-time PCR

Total RNA was extracted from the Isogen II solution and was transcribed using ReverTraAce qPCR RT Master Mix with gDNA Remover (TOYOBO). cDNA was used for Real-time PCR using Power SYBR Green Master Mix (Thermo Fisher Scientific) with the specific primers on a LightCycler 480 system II (Roche). Levels of mRNA expression were normalized relative to TATA-binding protein (*Tbp)* mRNA as an internal control using ∆∆Ct method. Sequences for the primers are shown in Supplementary Table 1.

### 2.4 Cloning of promoter region of human megalin gene

The -3996, -3135, -2071, -806, -539, -104/+200 fragments from the transcription start site of the human megalin promoter were amplified with genomic DNA from A549 cells by PCR and cloned into the luciferase reporter vector, pGL4.11 (Promega). Mutations were introduced into the HNF4α binding sites in the megalin promoter by inverted PCR based site-directed using the primers, 5′-**agggt**tgcagggggcgggccgggcg-3′ and 5′-cagcgcggggaggagtgggcactcgaa-3′. The induced mutations at -1/+4 in the megalin promoter are indicated as bold and underlined.

### 2.5 Transient transfection and luciferase assay

Wild-type, or mutated megalin promoters were cloned into pGL4.11, and pGL4.74 encoding *Renilla* luciferase as an internal control were co-transfected into HEK293T cells in the presence or absence of HNF4α expression plasmid. After 48 h, promoter activities were measured using Dual-Glo Luciferase Assay System (Promega).

### 2.6 Western blot

Whole cell lysates from cultured cell lines were also prepared as described previously [8]. The samples were diluted with Laemmli sample buffer, incubated at 65 °C for 15 min, fractionated by 10% SDS-polyacrylamide gel electrophoresis. The gels were transfered onto a PVDF membrane (GE healthcare). The membrane was incubated for 1 h with PBS containing 0.1% Tween 20% and 5% skim milk, and then incubated for 1 h with anti-HNF4α (Perceus Proteomics) and anti-γ-tubulin (Sigma-Aldrich) antibodies. After washing, the membrane was incubated for 1 h with horseradish peroxidase-conjugated secondary antibodies (Cell Signaling Technology), and the reaction product was visualized using Western Lightning Ultra (PerkinElmer).

### 2.7 Electrophoretic mobility shift analysis

Electrophoretic mobility shift analysis was carried out using LightShift Chemiluminescent EMSA kit (Thermo Fisher Scientific) and nuclear extracts from HNF4α-transfected cells. The following double-stranded probes were used (mutations are indicated as bold and underlined); the HNF4α binding site at -13/+17 in the human megalin promoter (wild-type (WT); 5′-ctccccgcgctgcaaagtgcagggggcggg-3′ and 5′-cccgccccctgcactttgcagcgcggggag-3′, mutant (Mut); 5′-ctccccgcgctg**agggt**tgcagggggcggg-3’ and 5’- cccgccccctgca**accct**cagcgcggggag-3’) and the HNF4α binding site at -203/-192 in the mouse ornithine transcarbamylase (*Otc*) promoter as a positive control (5′-gttaggcttaaagttcaagtg-3′ and 5′-cacttgaactttaagcctaac-3′) [7]. Nuclear extracts (3 μg) and the 5′-biotin labeled probe of the megalin promoter (WT) were added and the reaction mixture incubated on ice for 10 min. For competition experiments, a 50-fold excess of the unlabeled megalin (Mut) or *Otc* probes was added to the reaction mixture and the mixture was incubated on ice for 10 min prior to the addition of the 5′-biotin labeled probe. For supershift analysis, 1 μg of anti-HNF4α or anti-PPARβ antibodies (Santa Cruz Biotechnology) was added to the reaction mixture, and the mixture was incubated on ice for 10 min after the addition of the 5′-biotin labeled probe. DNA-protein complexes were fractionated by 7% PAGE, and blotted onto a Hybond-N^+^ membrane (GE healthcare). After washing, DNA-protein complexes were visualized using detection module in the kit.

### 2.8 Chromatin immunoprecipitation

HNF4α-transfected HEK293T cells were fixed in 1% formaldehyde for 10 min at room temperature and chromatin immunoprecipitation was carried out using SimpleChIP Plus Enzymatic IP kit (Cell Signaling Technology) and anti-HNF4α antibody (Santa Cruz Biotechnology). Purified DNA was amplified by real-time PCR using ΔΔCt method. Enrichment of the HNF4α binding site was normalized to the input samples and expressed as fold-enrichment as compared to the control normal IgG antibody. Nucleotide sequences of the primers are as follows: megalin gene with HNF4α binding site (5′-ggggttcagtaatcggaaga-3′ and 5′-gtgacaggacagcgaggtg-3′), megalin gene without HNF4α binding site (5′-ggggttcagtaatcggaaga-3′ and 5′-gtgacaggacagcgaggtg-3′), *OTC* gene with HNF4α binding site (5′-aaatgaggaggccaggcaa-3′ and 5′-ggttagagatactgcagggca-3′), and *OTC* gene without HNF4α binding site (5′-tggcaataccacactgtttagt-3′ and 5′-ctgaaccacaaggaccccaa-3′).

### 2.9 Statistical analysis

All values are expressed as the mean ± standard derivation (S.D.).

## 3 Results

### 3.1 Induced expression of proximal tubule-enriched genes by HNF4α

**Fig. 1:**
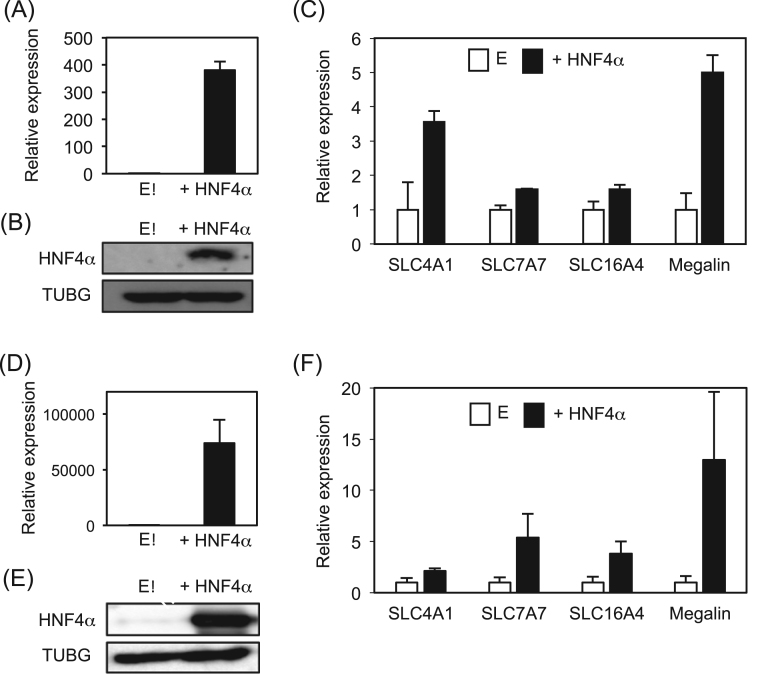
Expression of proximal tubule-enriched genes by HNF4α. HNF4α expression vector was overexpressed in HK-2 (A-C) and HEK293T (D-F) cells for 48 h. Real-time PCR for *HNF4A* (A and D) and Western blot of HNF4α and γ-tubulin (TUBG) (B and E). Real-time PCR for SLC transporter and megalin (C and F). *TBP* expression was used as an internal control to normalize expression of the target genes. Relative expression ± S.D. is presented as expression levels based on empty vector-transfected control (n = 3). E, empty vector.

HNF4α upregulates many genes in liver, pancreas, small intestine, and colon [3], [6], [7], [8], [9], [10], [12]. PTECs also highly express HNF4α, but involvement in PTEC function by HNF4α has not been investigated in detail. Thus, we analyzed to identify novel HNF4α target genes that are highly expressed in PTECs. By overexpression of HNF4α in human PTEC-derived HK-2 cells, we investigated whether HNF4α has a potential to induce the expression of proximal tubule-enriched genes. When HNF4α expression vector was transfected into HK-2 cells, HNF4α mRNA and protein was strongly induced (Fig. 1A and B). Then, we compared expression of 82 genes including transporters, ion channels, and receptors that are highly expressed in the proximal tubules. Of these, expression of 78 genes was induced 0.5–1.5-fold, or no expression by HNF4α (Supplementary Table 2). Expression of 4 genes including *SLC4A1*, *7A7*, *16A4*, and megalin was increased more than 1.5-fold by HNF4α (Fig. 1C). Similar results were observed in HEK293T cells (Fig. 1D-F). In rat kidney, HNF4α was shown to bind to *Slc22a1, Slc22a6,* and *Slc22a8* promoters [20], but the expression of these genes was not induced by HNF4α, or no expression in HK-2 cells (Supplementary Table 2). Of these, a noteworthy result was that expression of megalin was markedly induced more than 5- and 15-fold by HNF4α in HK-2 and HEK293T cells, respectively (Fig. 1C and F). Megalin plays an important role in reabsorption of albumin and low molecular weight proteins by endocytosis [23]. Interestingly, because megalin is invovled in obesity/metabolic syndrome-related CKD [16], we investigated whether HNF4α directly transactivates the expression of megalin.

### 3.2 Transactivation of megalin promoter by HNF4α

**Fig. 2:**
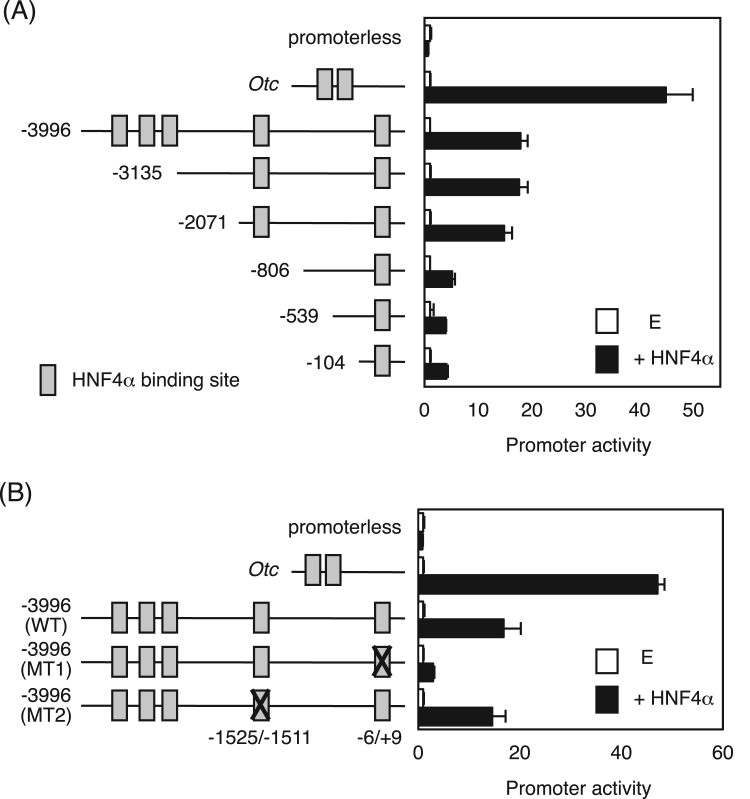
Promoter analysis of the megalin gene. The mouse *Otc* and the human megalin promoter constructs were co-transfected into HEK293T cells with empty vector or HNF4α expression vector. (A) Promoter activity of the deletion mutants of the megalin gene. (B) Mutations were introduced into the HNF4α⎕binding sites at -6/+9 (MT1) and at −1525/−1511 (MT2) and the constructs were co-transfected into HEK293T cells with HNF4α expression vector. The normalized activity is presented as relative activity based on promoterless vector. Data are mean ± S.D. (n = 3).

By the JASPAR, an open access database for transcription factor binding sites, five HNF4α binding sites with high score were expected in the human megalin promoter region from -4000 to +200 from the transcription start site. Thus, promoter analysis was performed to determine whether HNF4α can transactivate the megalin promoter (Fig. 2A). The mouse ornithine carbamylase (*Otc*) gene containing functional two HNF4α binding sites was strongly transcativated by HNF4α [7]. The megalin promoters of -3996/+200 fragment containing five HNF4α binding sites and -3135 and -2071/+200 fragments containing two HNF4α binding sites at -1525/-1511 and -6/+ 9 were transactivated by approximately 15–20 fold by HNF4α (Fig. 2A). The -806/+200 fragment containing only one HNF4α binding site at -6/+9 was still transactivated by HNF4α, but the promoter activity was decreased by approximately 5-fold, indicating that two regions at -1525/-1511 and -6/+9 might be essential for transactivation of the megalin gene by HNF4α. Thus, mutations were introduced into these HNF4α binding sites, resulting that the promoter activity of the mutant at -6/+9 (MT1) was decreased by approximately 80%, but the mutant at -1525/-1511 (MT2) had no significant difference in the promoter activity compared with wild-type promoter (Fig. 2B). These data indicate that the region at -6/+9 of the megalin promoter is a critical *cis*-element for transactivation by HNF4α.

### 3.3 Binding of HNF4 α to the megalin promoter

**Fig. 3:**
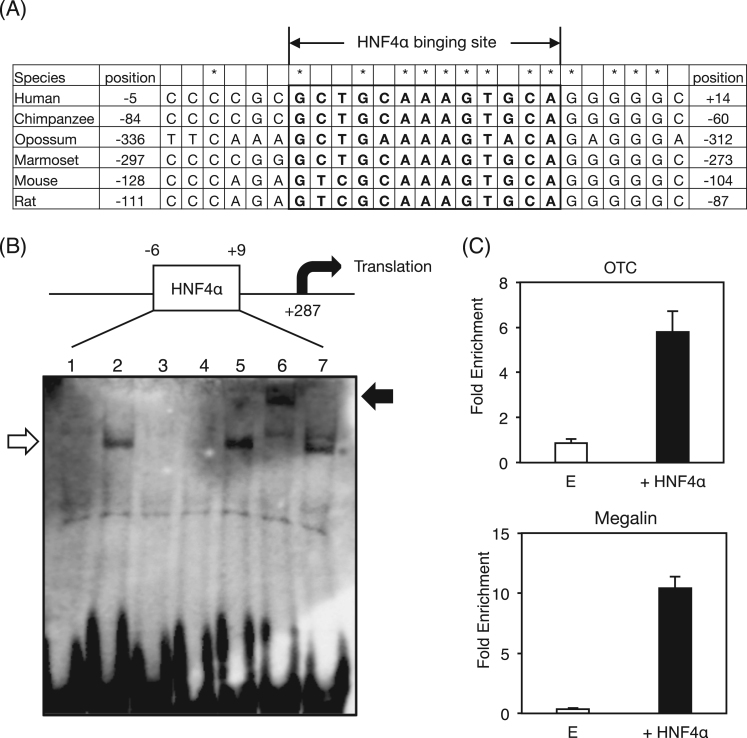
Binding of HNF4α to the megalin promoter. (A) Sequence alignment of the megalin promoter in human, chimpanzee, opossum, marmoset, mouse, and rat. Predicted HNF4α⎕binding site is enclosed in box. Completely conserved nucleotides among the species are shown as asterisk. (B) Electrophoresis mobility shift assay. The megalin probe carrying the HNF4α binding site at 6-/+9 was incubated without or with nuclear extracts from HNF4α-transfected HEK293T cells (lanes 1 and 2). The megalin probe with the nuclear extracts was incubated with the nuclear extracts in the presence of excess amount of the unlabeled *Otc* competitor (lane 3), the unlabeled megalin competitor (lane 4), and the unlabeled megalin competitor containing the mutated HNF4α⎕binding site (lane 5). For supershift analysis, anti-HNF4α and anti-PPARβ antibodies were added (lanes 6 and 7). (C) Chromatin immunoprecipitation using empty vector or HNF4α expression vector-transfected HEK293T cells were performed with anti-HNF4α antibody and normal goat IgG. The regions with or without the HNF4α binding sites in the human *OTC* and megalin gene were amplified. The data from real-time PCR was normalized relative to the input and expressed as fold-enrichment over the data from IgG control. Data are mean ± S.D.

The binding site at -6/+ 9 of the megalin gene was highly conserved among species (Fig. 3A). To confirm the direct binding of HNF4α at -6/+9 in the megalin promoter, electrophoresis mobility shift analysis was performed (Fig. 3B). Nuclear extracts from HNF4α-transfected HEK293T bound to biotin-labeled probe including the -6/+9 fragment of the megalin promoter (lane 2, the lower open arrow). This complex band was eliminated by the addition of excesses of unlabeled probe containing an HNF4α binding site in the mouse *Otc* promoter (lane 3) and the unlabeled megalin probe containing the -6/+9 fragment (lane 4), but not the unlabeled megalin probe whose mutations were introduced into the HNF4α binding site at the -6/+9 region (lane 5). The complex was supershifted by anti-HNF4α antibody, not by anti-PPARβ antibody (lanes 6 and 7, the upper closed arrow), indicating that HNF4α directly binds to the megalin promoter. Furthermore, chromatin immunoprecipitation analysis using HNF4α-transfected HEK293T cells showed that HNF4α indeed bound to the HNF4α binding sites of the *OTC* promoter as the positive control (Fig. 3C). HNF4α strongly bound to the megalin promoter region, suggesting that HNF4α directly and physiologically binds to the promoter region of the megalin gene.

## 4 Discussion

HNF4α is a master regulator in liver, and many function and the target genes of HNF4α have been identified [7], [8], [9]. Similarly, function of HNF4α in pancreatic β-cells and intestinal epithelial cells was investigated [10], [12]. HNF4α also highly expresses in PTECs in kidney, but function of HNF4α in the PTECs has been poorly investigated due to lack of proximal tubule-specific *Hnf4a*-null mice. Thus, we aimed to identify novel HNF4α target genes that are highly expressed in the PTECs by HNF4α overexpression system.

Expression of 3 SLC transporters for chloride/bicarbonate (SLC4A1) [24], cationic amino acids/neutral amino acids (SLC7A7) [25], and orphan monocarboxylate (SLC16A4) [26], was upregulated more than 1.5-fold by HNF4α in human PTEC-derived HK-2 cells, these upregulated SLC transporters may be the novel target genes for HNF4α in the PTECs. HNF4α binds to *Slc22a1, Slc22a6,* and *Slc22a8* promoters in rat kidney [20], but the expression of these genes was not induced by HNF4α, or no expression in HK-2 cells. Thus, HNF4α only binds to these promoters, and transactivation of these genes may not be induced in human PTECs.

Interestingly, expression of megalin was strongly induced by HNF4α. Megalin was first discovered as the pathological antigen of Heymann nephritis in the rat proximal tubule and cloned as gp330/megalin that is a large glycoprotein belonging to LDL receptor superfamily [27], [28]. Subsequently, megalin was found to be a receptor for a large number of ligands such as calcium ion, retinol-binding proteins, vitamin D-binding proteins, albumin, transthyretin, and liver-type fatty acid binding protein [29]. Whole body megalin-null mice indeed result in low molecular weight proteinuria of serum carrier proteins such as retinol-binding proteins and vitamin D-binding proteins, and the similar symptoms occur in Fanconi syndrome [30]. Kidney-specific megalin-deficient mice also exhibit decreased plasma vitamin D, probably due to decreased uptake of vitamin D-binding protein/25-OH vitamin D_3_ complex, and result in hypocalcemia and severe osteopathy, suggesting that megalin is a critical receptor for calcium homeostasis and bone metabolism [31]. Mutations in megalin gene were found in families with Donnai-Barrow syndrome and facio-oculo-acoustico-renal syndrome, revealing that megalin is an important regulator of many compounds in the bloodstream [32]. Interestingly, PTECs in high-fat diet fed wild-type mice, but not kidney-specific megalin-null mice, showed autolysosomal dysfunction with autophagy impairment, indicating that megalin might be associated with CKD development and progression [33].

Although transcriptional regulation of megalin have not been investigated in detail, it was reported that peroxisome proliferator-activated receptor α (PPARα) and γ (PPARγ) positively regulates the expression of megalin through the binding sites in the promoter region [34]. However, expression of megalin was only about 2-fold induced by the ligands for PPARs, indicating that other transcription factors synergistically regulate expression of megalin gene with PPARα/γ. Because HNF4α induced megalin expression about 15-fold, HNF4α may be an important factor to transativate the megalin gene in PTECs. Several studies have reported that HNF4α and PPARα could share the binding sites in the promoter regions [35]. For example, HNF4α and PPARα bind to the same sequence in acyl-CoA thioesterase 1 (*Acot1*) promoter [36]. In the report, expression of *Acot1* was upregulated by treatment of ligand for PPARα, but the expression of *Acot1* is repressed in the presence of HNF4α. Conversely, PPARα was shown to bind to multiple binding sites in the megalin promoter [34], but no transactivation by HNF4α was observed through these binding sites in this study. HNF4α bound to the other region including the transcription start site and transactivated the expression of megalin, indicating that HNF4α and PPARα may positively regulate the expression of megalin though different binding sites in PTECs. Because saturated and unsaturated free fatty acids and their derivatives are endogenous ligands for PPARα [37] and megalin uptakes albumin-bound fatty acids into PTECs, PPARα might be transactivated by these fatty acids uptaken through megalin. Because of expression of PPARα is positively regulated by HNF4α [38], HNF4α may play a dual role in megalin expression through direct regulation of megalin itself and indirect regulation of megalin by PPARα.

In conclusion, HNF4α was found to upregulate many genes including transporters and megalin that are highly expressed in PTECs. Of these, HNF4α strongly upregulated megalin expression through an HNF4α binding site in the promoter region. Because dysfunction of transporters and megalin causes many diseases including CKD, HNF4α may play a critical role in maintaining the function of PTECs.

## Funding

This work was supported in part by grants from the Ministry of Education, Culture, Sports, Science, and Technology of Japan (Grant–in-Aid for Scientific Research, No. 25460490).

## Conflict of interest

The authors declare no conflicts of interest associated with this manuscript.
