# Supplementary material for: Hepatocyte nuclear factor 4α regulates megalin expression in proximal tubular cells
Source: Biochem Biophys Rep. 2018 Dec 12;17:87–92. doi: 10.1016/j.bbrep.2018.11.010 (PMC6295598; doi:10.1016/j.bbrep.2018.11.010)
Supplement: Supplementary file 2 — Supplementary material [file mmc2.pdf]

Supplementary Table 1. Primers used in quantitative RT-PCR

Gene	Nucleotide sequence		Gene	Nucleotide sequence	
human	F	accctgatcattgctgtcg	human	F	agtgactccatccctggactt
SLC1A1	R	ggagagcttttccacaatgc	*SLC16A2*	R	ggggaatcatcatggacatc
human	F	gtcaacacggccttcactg	human	F	gagtttgggatcggctacag
SLC2A1	R	ggtcatgagtatggcacaacc	*SLC16A3*	R	cggttcacgcacacactg
human	F	ttatctgcattggcgtgttc	human	F	tctttgtgtgactatgggacttct
SLC2A9	R	gcagggaccacaatcactc	*SLC16A4*	R	gctgtagaaagagccaatcgtt
human	F	tggaacgatggaagattttga	human	F	aacggtcccctgagatcat
SLC3A1	R	cactcgtgtggtttggtatga	*SLC16A5*	R	ccactcatggctggtcct
human	F	tcttcaggaacgtggagctt	human	F	tccaatagcaggatctcatgg
SLC4A1	R	ggccacttcgtcgtattcat	*SLC16A6*	R	gaaacgattgctcaggactgt
human	F	gccaataattgcatttgtgaga	human	F	tgtggcccagttcttcttgg
SLC4A7	R	ccgctggacccaataaca	*SLC16A7*	R	tgctgctaccacaatagccc
human	F	gccattggaggctttgaa	human	F	ctggtgttgctcgtggagg
SLC5A1	R	caccaccccagccttaatatag	*SLC16A8*	R	ccggccaggtagaagatgatc
human	F	tgctgacatcctagtcattgct	human	F	gcttggcctgatttcaaca
SLC5A2	R	tgttggttctgcacatggac	*SLC16A9*	R	gcatccatccagtccatagaa
human	F	ccaggactttttgtggcttg	human	F	ttgggctcatgtccagttct
SLC5A12	R	aaaggtcactgttgccaagg	*SLC16A10*	R	accaatgaaggctggtatgc
human	F	cgccttcgtgtgtagcatc	human	F	cgtattcggggctctgac
SLC6A6	R	gctgcatagtagtcaaagagctga	*SLC16A11*	R	tcagcccataggccacag
human	F	tgggaggtgaccctttgtc	human	F	gcagtgccatacttggtgag
SLC6A8	R	cgtaggggaatgtagcagtga	*SLC16A12*	R	caaagccaagcaacacagaa
human	F	tcccctacctgtgtcagagc	human	F	tgcagatgatagagagcatcg
SLC6A19	R	gcgaactccaggtacagca	*SLC16A13*	R	gaagccgtgtagttgcctgt
human	F	ccagtgccttctctgctctt	human	F	ctatgctgcaaacgtgcatt
SLC7A7	R	agccccacaaagaaccagta	*SLC16A14*	R	gaaatacctgcccaccatga
human	F	catgaacgtgtcctacttcacc	human	F	acttgtgactggagttatctgtgaa
SLC7A9	R	aacacggtcaccaaatgtca	*SLC17A1*	R	agagaagacatacggcacagc
human	F	tcttcaccgtctttgtgcag	human	F	gggtggccctttgtcttc
SLC9A1	R	atggagcgcttcgtctctt	*SLC17A3*	R	ggaaacggggtcatcataaa
human	F	ctgaaggatgccatcagctac	human	F	cagttcctcgtgcccatc
SLC9A3	R	gaccacgttgtcggtgct	*SLC19A1*	R	ggcaaagaacgtgttgacc
human	F	caatggggagatacagaaggag	human	F	ccttcctgaccccgtacc
SLC9A3R1	R	ctcgctggtgtcactggag	*SLC19A2*	R	agcaccaggtaagagtaagtcca
human	F	cccagtcacccagatcctc	human	F	aaaacctgaccagtgcagaga
SLC9A8	R	aggccaagaaatgcaaacac	*SLC19A3*	R	ggacataatcggtgaggacaa
human	F	tggtgctcattataggatgctg	human	F	catggggaaggacctcact
SLC10A2	R	gcagtgtggagcatgtggt	*SLC20A2*	R	ggaggcgatcaccactgt
human	F	ctcgacactggctgtggac	human	F	tcaagtggtattaaaaagcatacagtg
SLC11A2	R	ccccactgcccaaatgta	*SLC21A8*	R	ttcacccaagtgtgctgagta
human	F	tgctttacttatagggctgctattc	human	F	ctggaggctttctgcttcac
SLC13A1	R	gtggagagtaatcaaaagcaacaa	*SLC21A20*	R	gcttctcaacagtggaaatgcta
human	F	ctccatggcctttgtggt	human	F	tcctcttcctgctctactactgg
SLC15A1	R	aactttaatttggacttcgtttcc	*SLC22A1*	R	tggtccattatctttattgcttca
human	F	caggtcttgagttttcttattctcag	human	F	tcggctacatagcagacagg
SLC15A2	R	cgatgatattcccaactgcaa	*SLC22A2*	R	cgtataggttggggaaatgg
human	F	gggattggtgaccattgtg	human	F	gtgctgccactgtttgctta
SLC16A1	R	catgtcattgagccgacct	*SLC22A5*	R	gggggactcagggatgaa

Gene	Nucleotide sequence		Gene	Nucleotide sequence	
human *SLC22A6*	F<br>R	gctggggaagggttgtct<br>attcccatgcctgtctgc	human *SLC34A3*	F<br>R	gaaatccatgccgagttcc<br>cctccaagactggagcagaa
human *SLC22A7*	F<br>R	gatggggaaagctttttctga<br>agccccatccctgtctgt	human *SLC34A3*	F<br>R	gaaatccatgccgagttcc<br>cctccaagactggagcagaa
human *SLC22A8*	F<br>R	gctcgtgcttggagacct<br>ccatgtagatggggaaggtg	human *SLC40A1*	F<br>R	gctctagctgtgaaagctggtc<br>agttccctccaggggtttt
human *SLC22A11*	F<br>R	ttccgctccctgacactatc<br>acagtgacggcctcttgc	human *MATE1*	F<br>R	gtgctctttgctgtagccttc<br>cctgagccaccagattaatga
human *SLC22A12*	F<br>R	ggattctggtgggagctg<br>tacccatcacagccatctga	human *MATE2-K*	F<br>R	tatactgcccggctggact<br>tgcagtgctctctgctctct
human *SLC22A13*	F<br>R	atgagacgcagccttgtga<br>cgatcacaaaccaggttgaa	human *AQP1*	F<br>R	cctccctgactgggaactc<br>ggagggtcccgatgatct
human *SLC23A3*	F<br>R	tggactctcccccaggtt<br>tccagcagacaaaaccacag	human *AQP7*	F<br>R	gggttttggcttcggagt<br>cgcacagttagcaaaggtca
human *SLC24A3*	F<br>R	gtgtctgtgggctctttgct<br>cgatcacagacagcgtgtagta	human *AQP11*	F<br>R	ggctgcactcatcaccttttt<br>aatgtagcgaaagtgccaaag
human *SLC25A8*	F<br>R	tgaaagccaacctcatgaca<br>ctacaggggaggcgatgac	human *KCNE1*	F<br>R	tgtatccagaggaaatagccaag<br>ggatcatcctgggcattaag
human *SLC26A1*	F<br>R	gatgaccgggctttaccag<br>ctgcgaggtcaggatggt	human *PKD1*	F<br>R	agcctgaccgtgtggaag<br>ggacacacactccaaggaca
human *SLC26A2*	F<br>R	ggttggcagcactgtaacct<br>gacagaaacaaaacccacttga	human *LRP2*	F<br>R	atagaggggagcaccactga<br>agcaatttcctccgtgcat
human *SLC26A3*	F<br>R	tcggcacttccagacacata<br>gcgatctgggactgcttttt	human *BMP7*	F<br>R	tccaagacgcccaagaac<br>acagctcgtgcttcttacagg
human *SLC26A4*	F<br>R	cattgttaaatccatcccaagg<br>tgcaatagcataagccacca	human *ANG*	F<br>R	cattgtcctgcccgtttc<br>cagcacgaagaccaacaaca
human *SLC28A1*	F<br>R	catgctgggaggcttgac<br>cacaggctcccgtgaaga	human *PDGFD*	F<br>R	tgtggctaaacctggattcaa<br>tcccagttggtctctgaagc
human *SLC28A2*	F<br>R	caggggagctgaagctga<br>ggttggtgccattcagaga	human *CUBN*	F<br>R	ccgttagtgacgacaaggtg<br>ccaggtagtcatgggagca
human *SLC29A1*	F<br>R	ccttctccaacggctacct<br>cacaggaagaaggccatgat	human *AMN*	F<br>R	gtgctgtgacctctgtggag<br>acccaggaaggtgtccagta
human *SLC29A2*	F<br>R	acggagcctgacctcttactt<br>gaagaggggcacgaacag	human *HNF4A*	F<br>R	gtgctgtgacctctgtggag<br>acccaggaaggtgtccagta
human *SLC34A1*	F<br>R	gctccagcacctccacat<br>atgttggagcccatgatga	human *TBP*	F<br>R	gaacatcatggatcagaacaaca<br>atagggattccgggagtcat
