# Supplementary material for: Hepatocyte nuclear factor 4α regulates megalin expression in proximal tubular cells
Source: Biochem Biophys Rep. 2018 Dec 12;17:87–92. doi: 10.1016/j.bbrep.2018.11.010 (PMC6295598; doi:10.1016/j.bbrep.2018.11.010)
Supplement: Supplementary file 3 — Supplementary material [file mmc3.pdf]

Supplementary Table 2. Expression of proximal tubule-enriched genes

Gene	Empty	+ HNF4α	Gene	Empty	+ HNF4α	Gene	Empty	+ HNF4α
SLC1A1	1.00±0.37	1.17±0.12	*SLC16A7*	1.00±0.09	0.86±0.14	*SLC24A3*	N.D.	N.D.
SLC2A1	1.00±0.18	1.11±0.04	*SLC16A8*	N.D.	N.D.	*SLC25A8*	1.00±0.20	1.00±0.08
SLC2A9	1.00±0.05	1.06±0.05	*SLC16A9*	1.00±0.24	1.08±0.29	*SLC26A1*	1.00±0.18	1.31±0.44
SLC3A1	1.00±0.68	0.80±0.25	*SLC16A10*	1.00±0.23	1.05±0.04	*SLC26A2*	1.00±0.10	0.92±0.16
SLC4A7	1.00±0.10	0.96±0.06	*SLC16A11*	1.00±0.45	1.24±0.14	*SLC26A3*	N.D.	N.D.
SLC5A1	N.D.	N.D.	*SLC16A12*	1.00±0.34	1.09±0.10	*SLC26A4*	N.D.	N.D.
SLC5A2	N.D.	N.D.	*SLC16A13*	1.00±0.17	1.16±0.11	*SLC26A6*	1.00±0.08	1.24±0.06
SLC5A12	N.D.	N.D.	*SLC16A14*	1.00±0.45	0.59±0.19	*SLC28A1*	N.D.	N.D.
SLC6A6	1.00±0.03	0.97±0.08	*SLC17A1*	1.00±0.21	0.87±0.52	*SLC28A2*	N.D.	N.D.
SLC6A8	1.00±0.19	1.07±0.10	*SLC17A3*	1.00±0.04	1.01±0.31	*SLC29A1*	1.00±0.16	1.13±0.04
SLC6A19	N.D.	N.D.	*SLC19A1*	1.00±0.20	1.05±0.09	*SLC29A2*	1.00±0.27	1.08±0.16
SLC7A9	1.00±0.54	0.87±0.31	*SLC19A2*	1.00±0.17	1.16±0.08	*SLC34A1*	N.D.	N.D.
SLC9A1	1.00±0.15	1.08±0.12	*SLC19A3*	N.D.	N.D.	*SLC34A3*	1.00±0.27	0.74±0.18
SLC9A3	1.00±0.40	0.63±0.24	*SLC20A2*	1.00±0.06	1.23±0.06	*SLC40A1*	1.00±0.11	1.13±0.35
SLC9A3R1	1.00±0.20	1.33±0.05	*SLC21A8*	N.D.	N.D.	*MATE-1*	1.00±0.59	0.90±0.46
SLC9A8	1.00±0.06	0.97±0.19	*SLC21A20*	N.D.	N.D.	*MATE2-K*	1.00±0.10	0.93±0.49
SLC10A2	N.D.	N.D.	*SLC22A1*	1.00±0.02	1.30±0.22	*AQP1*	N.D.	N.D.
SLC11A2	1.00±0.06	1.15±0.07	*SLC22A2*	1.00±0.44	1.36±0.28	*AQP7*	1.00±0.50	1.33±0.13
SLC13A1	N.D.	N.D.	*SLC22A5*	1.00±0.07	1.22±0.06	*AQP11*	1.00±0.05	1.07±0.04
SLC15A1	1.00±0.64	0.95±0.53	*SLC22A6*	N.D.	N.D.	*KCNE1*	N.D.	N.D.
SLC15A2	1.00±0.30	0.99±0.30	*SLC22A7*	N.D.	N.D.	*PKD1*	1.00±0.08	0.86±0.21
SLC16A1	1.00±0.31	1.16±0.15	*SLC22A8*	1.00±0.41	0.86±0.21	*BMP7*	1.00±0.50	1.20±0.41
SLC16A2	1.00±0.08	1.34±0.18	*SLC22A11*	N.D.	N.D.	*ANG*	1.00±0.21	0.99±0.05
SLC16A3	1.00±0.24	0.72±0.09	*SLC22A12*	N.D.	N.D.	*PDGFD*	N.D.	N.D.
SLC16A5	1.00+0.17	1.10±0.03	*SLC22A13*	1.00±0.09	0.63±0.48	*CUBN*	1.00±0.10	0.91±0.22
SLC16A6	1.00±0.63	0.85±0.14	*SLC23A3*	1.00±0.06	1.43±0.34	*AMN*	1.00±0.47	1.02±0.36

N.D., not detected.
